# Simulating Irrational Human Behavior to Prevent Resource Depletion

**DOI:** 10.1371/journal.pone.0117612

**Published:** 2015-03-11

**Authors:** Anna Sircova, Fariba Karimi, Evgeny N. Osin, Sungmin Lee, Petter Holme, Daniel Strömbom

**Affiliations:** 1 Department of Psychology, Umeå University, Umeå, Sweden; 2 Independent researcher, Copenhagen, Denmark; 3 IceLab, Department of Physics, Umeå University, Umeå, Sweden; 4 International Laboratory of Positive Psychology of Personality and Motivation, National Research University Higher School of Economics, Moscow, Russia; 5 Department of Energy Science, Sungkyunkwan University, Suwon, Korea; 6 Department of Mathematics, Uppsala University, Uppsala, Sweden; 7 Department of Biology, Lafayette College, Easton, Pennsylvania, United States of America; Tianjin University of Technology, CHINA

## Abstract

In a situation with a limited common resource, cooperation between individuals sharing the resource is essential. However, people often act upon self-interest in irrational ways that threaten the long-term survival of the whole group. A lack of sustainable or environmentally responsible behavior is often observed. In this study, we examine how the maximization of benefits principle works in a wider social interactive context of personality preferences in order to gain a more realistic insight into the evolution of cooperation. We used time perspective (TP), a concept reflecting individual differences in orientation towards past, present, or future, and relevant for making sustainable choices. We developed a personality-driven agent-based model that explores the role of personality in the outcomes of social dilemmas and includes multiple facets of diversity: (1) The agents have different behavior strategies: individual differences derived by applying cluster analysis to survey data from 22 countries (N = 10,940) and resulting in 7 cross-cultural profiles of TP; (2) The non-uniform distribution of the types of agents across countries; (3) The diverse interactions between the agents; and (4) diverse responses to those interactions in a well-mixed population. As one of the results, we introduced an index of overall cooperation for each of the 22 countries, which was validated against cultural, economic, and sustainability indicators (HDI, dimensions of national culture, and Environment Performance Index). It was associated with higher human development, higher individualism, lower power distance, and better environmental performance. The findings illustrate how individual differences in TP can be simulated to predict the ways people in different countries solve the personal vs. common gain dilemma in the global limited-resource situation. This interdisciplinary approach to social simulation can be adopted to explain the possible causes of global environmental issues and to predict their possible outcomes.

## Introduction

The need for sustainability was clearly articulated back in 1987, when Gro Harlem Brundtland, a former prime minister of Norway, presented her report [1], which defined sustainable development as development that meets the needs of the present generation without compromising the ability of future generations to meet their own needs. However, resource depletion is our everyday reality. By 2030, there will be three billion more middle-class consumers in the global economy; on current trends, over the next 20 years we will use 40% more water than we do now; the average cost of drilling for oil has doubled over the past decade [2] and the Earth has lost about 1,429,098 square km of forest in the last 12 years [3].

The overall situation resembles more and more the ‘Tragedy of the Commons’ [4], a classical social dilemma. It is a situation in which a shared resource is being overharvested and depleted by rational, utility-maximizing individuals unwilling to cooperate and sacrifice their own comfort in the short term for the long-term benefit of all. The level of awareness of environmental problems is not the same in different countries [5,6] and countries exhibit vast differences in the implementation of environmentally friendly practices. However, with respect to our over-harvesting of the Earth’s resources, the awareness of the problem is not enough to bring about changes in our actions. In order to understand the potential mechanisms of change, we need to explore the ways the attitudes, norms, and behaviors of individuals translate to the outcomes of social dilemmas at the group level.

The social dilemma research has undergone interesting developments in the recent years (see [7] for review). The solutions depend on psychological or motivational factors: social values endorsed by individuals, communication (information about others’ choices or moral persuasion), presence of a group identity, group reciprocity, payoff structure, actual or perceived efficacy of individual choice (presence of feedback of choice consequences), group size, presence of boundaries (e.g., regulation of access to the resource) and sanctions for their transgression (e.g. overuse) [8, 9]. Several theoretical frameworks are developing in the field. Interdependence theory describes the ways psychological factors (self-interest, altruism, collectivism, egalitarianism, etc.) influence subjective evaluation of outcomes and cooperative behavior in situations of social or temporal conflict. Appropriateness framework stresses “that features of the objective situation impact the decision maker’s identity and how the situation is perceived;… identity is driven by a decision maker’s personal history (e.g., individual differences, learning). … the model stresses decision makers’ construal of the situation” [7], p.128. Studies within the public goods game (PGG) paradigm have explored the way personal expectations for the environment affect cooperation and agglomeration [10], the impact of heterogeneity of investment in cooperation [11], and the role of individual diversity of agents in a spatial PGG with two types of players in a lattice [12].

However, there is no single theoretical framework to address the impact of individual differences between decision makers on the social dilemma outcomes. Most studies have used experimental paradigm, focusing mainly on situational factors and on the effects of stable individual differences addressed by studying contrast groups of individuals. However, a real-life social dilemma involves a complex interaction between individuals with different values and strategies that affect each other in various ways. The composition of the group (the percentage of people with certain psychological characteristics) may affect the outcome of interaction for the group as a whole [13]. Because this interaction is a dynamic process involving a large number of independent agents with different properties, the outcome of social dilemma can be seen as a dynamic characteristic of the system that evolves with time. An approach that tries to predict this characteristic as a linear function of the average characteristics of individuals comprising a given group can be seen as overly simplistic. However, when it comes to explaining the causes of observed differences in pro-environmental behavior at the group level (e.g. in different countries), this approach prevails. Researchers working at the country level link pro-environmental attitudes to cultural values and socioeconomic variables, such as the GDP [5,6], without addressing individual differences. Researchers working at the individual level have discovered a range of psychological variables predicting pro-environmental behavior [14,15], but the way these diverse mechanisms form an integral behavioral strategy and the way individual strategies interact, resulting in group-level outcomes, are not yet clear.

In our study we challenge the usual rational agent approach. We view the decision makers in a situation resembling the ‘Tragedy of the commons’ as irrational agents building their behavior strategies and their interaction with other agents based on their personality traits. Previous studies focusing on individual differences in personality did not take into account the effect of the distribution of individuals with different traits within the population. Our approach can be placed within the appropriateness framework [16], which it expands. We advance the research field of social dilemmas by addressing the irrational character of the personality dimension and by taking into account the differences in the distribution of personality characteristics across populations

The present study provides an example of the way social simulation approach can be used to link individual-level psychological factors with group-level behavioral outcomes in a social dilemma situation, taking into account individual differences and the dynamics of human interaction. With this study we address the lack of studies of the group-level effects of interaction among the agents on social cooperation [17, 12]. We focus on heterogeneity of cooperation, which depends on the personality types of agents and use a larger number of personality types to model the diversity of individuals in a more accurate way.

In our approach we follow the tradition that stresses the role of the temporal dimension in social dilemmas, which are defined as “conflicts between short-term self-interest and long-term collective interest” [18], p.127; [7], p.125, and involve not only a conflict of values (should one act for one’s own best or for the common good with certain sacrifices on one’s part), but also a conflict of temporal perspectives (should one consider short-term or long-term consequences of one’s decisions). Individual differences in the consideration of future consequences predict decision-making in various social dilemmas and are associated with acting in pro-environmental ways and having pro-environmental attitudes [18,19]. At the group level, however, attempts to link long-term orientation as a dimension of national culture to the environmental performance of countries have produced inconclusive findings, some contradicting the individual-level associations [20].

Our study draws upon an extended understanding of temporal orientation as a set of orientations toward the past, present, and future, based on the classical Lewin’s psychological field theory [21] and the Zimbardo Time Perspective Inventory (ZTPI) [22]. The ZTPI is a multidimensional self-report instrument which has been validated in different cultures [23] and measures five dimensions of time perspective (TP): Past Negative (PN), reflecting a pessimistic, negative or aversive attitude toward the past; Past Positive (PP), a warm, sentimental, nostalgic, and positive outlook on the past; Present Hedonistic (PH), which reflects a hedonistic risk-taking attitude toward time and life; Present Fatalistic (PF), which embodies a helpless and hopeless attitude toward the future and life; and Future (F), a general future orientation, where one is striving for future goals and rewards.

Extensive research shows the associations between these dimensions of TP and numerous behaviors [24], including pro-environmental behavior [25]. The drawbacks of the existing studies of TP are their correlational nature and focus on isolated variables, which does not help to explain the way these dimensions of TP interact. Based on the five dimensions several integral personality profiles of TP were theoretically proposed. Successful attempts to operationalize them have been made, focusing mainly on the balanced time perspective profile [22,26,27]. The heuristic potential of focusing on integral personality types in understanding the principles underlying the dynamics of human behavior has been demonstrated in developmental psychology [28]. We set to explore this in our study.

In this study we model the way individual differences in time perspective affect group behavior and the outcome of a ‘tragedy of the commons’ social dilemma using the agent-based modeling approach (ABM). In contrast to other studies, we did not aim to examine the impact of behavioral strategies of individual agents *per se*, but rather to focus on the personality factor that can influence individual behavioral strategies and affect the overall outcome, the development or evolution of the complex system.

We see the purpose of simulation (just as that of more traditional research approaches) in application to complex human behavior not in reproducing the whole complexity of reality, but in isolating specific factors that may be pertinent to realistic situations and studying the consequences of individual differences and contextual effects. ABM can be seen as a thought experiment that becomes a feasible strategy in cases when the group-level outcomes are not easily predictable from individual-level associations, and the social systems are too large in scale to be reproduced with any sufficient approximation in real-life experimental settings [29]. In these models, agents’ interactions at the individual level typically produce emergent properties at the group level. ABM allows us to implement not only the rational strategies (like those described in game theory) in the model, but also interaction rules [30] and contextual influences, such as feedback effects of the state of the whole group on individual behavior.

One of the principal difficulties in simulating human behavior with ABM is the nature of individual differences and the way they are studied. Firstly, they are inherently complex and ambiguous, expressed in terms of linguistic constructs with rather vague definitions [31]. Thus, creation of agents for the simulation of personality processes involves building probabilistic models based on the principles of fuzzy logic [32]. Secondly, the majority of psychological studies are correlational, leaving open the question of the way specific constructs or phenomena reveal themselves in real-life behavior and affect the daily decision-making process. As a result, expert ratings or focus groups may be needed to combine and transform the existing body of psychological findings into a set of rules and algorithms that would reflect the essential aspects of relevant psychological phenomena without overly complicating the model. We describe our approach further in the methods section.

Our model was inspired by the story of a village in the Netherlands during a harsh winter in 1978.

Due to an unusually heavy snow, the small village was completely cut off from the rest of country so that there was no electricity to use for light, heating, television, etc. However, one of the 150 inhabitants owned a generator that could provide sufficient electricity to all people of this small community, if and only if they exercised substantial restraint in their energy use. For example, they should use only one light, they should not use heated water, the heating should be limited to about 18 Celsius, and the curtains should be closed. As it turned out, the generator collapsed because most people were in fact using heated water, living comfortably at 21 Celsius, watching television, and burning several lights simultaneously. After being without electricity for a while, the citizens were able to repair the generator, and this time, they appointed inspectors to check whether people were using more electricity than they agreed upon. But even then, the generator eventually collapsed due to overuse of energy. And again, all inhabitants suffered from the cold and lack of light, and of course, could not watch television [18], p.127–128.

We set up a model compatible with the story and used self-report data on time perspective available from 22 different countries^i^ [23]. We aimed to develop a methodology for including self-report data on individual differences in simulations and to model the way cooperation varies in different populations due to an interplay between personality types, their distribution, and social interactions between the agents.

## Methods

Our study had three stages. At Stage 1 we defined the TP profiles and their distribution in different countries. At Stage 2 we defined the behavior and interaction strategies for each type. At Stage 3 we built the simulation.

Stage 1: To specify the typical TP profiles, we used the dataset from the 22 countries (with a total of 10,940 participants). Individual time perspective was assessed by the ZTPI, a self-report measure of individual orientations and attitudes towards past, present and future with 56 items tapping the five TP dimensions. Participants indicated the extent to which statements were characteristic or true of them on a five-point Likert scale. Details of the sample and cross-cultural equivalence of the ZTPI can be found here [23].

To determine the typical TP profiles across countries and their distribution within each country sample we applied the person-oriented approach [28, 33] using hierarchical cluster analysis methodology. This holistic approach is particularly beneficial for the simulation studies based on nonlinear dynamic models of human behavior. It allows to classify individuals, resulting in a set of clusters with different meaningful combinations of psychological constructs, and to use qualitative analysis to infer hypotheses about the ways people belonging to each latent type may change over time and interact with one another.

**Fig 1:**
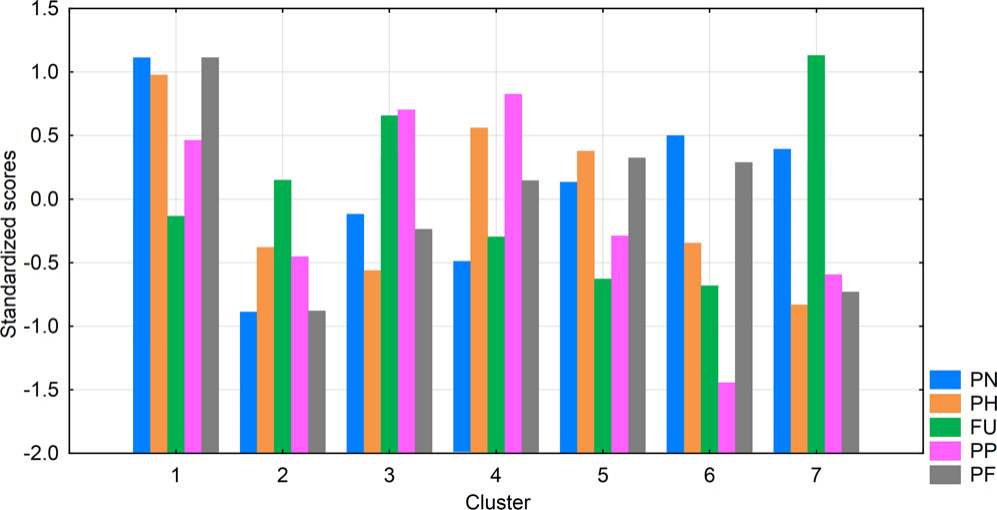
Standardized scores of the seven TP profiles on the ZTPI scales. Note: PN—Past-Negative, PH—Present-Hedonistic, FU—Future, PP—Past-Positive, PF—Present-Fatalistic.

We applied cluster analysis using Ward’s method with the Squared Euclidean distance metric to the cross-cultural dataset. The scores on the five ZTPI subscales were standardized within each country in order to emphasize individual differences and remove the bias resulting from potentially non-equivalent item intercepts between the countries [34]. Based on examination of the plot of variance explained [35] and on substantive analysis of different cluster models, we chose a seven-cluster model. The means of the ZTPI scale scores (shown on Fig. 1) in each cluster defined 7 meaningful TP profiles (or ‘personality types’).

Time perspective profiles were non-uniformly distributed across the 22 country samples (Pearson χ^2^ = 264.72, df = 138, p<0.001) with each profile sufficiently represented in each. We used these distributions (presented in Table 1) in our model.

**Table 1:** Cluster distribution per country (numbers indicate percentages of members of each cluster of the respective country sample).

	Clusters						
Country	1	2	3	4	5	6	7
Algeria	12.21	19.59	19.35	10.14	25.58	9.91	3.23
Brazil	15.91	20.64	21.59	10.98	17.61	7.77	5.49
China–1	15.34	21.02	23.86	10.23	16.48	10.51	2.56
China–2	15.80	21.97	20.24	9.74	18.40	11.36	2.49
Croatia	14.61	18.26	19.94	12.64	17.98	9.27	7.30
Czech Republic	14.41	18.29	21.47	8.95	23.86	4.67	8.35
France	12.17	18.14	22.43	12.17	19.81	8.35	6.92
Germany	11.21	17.29	19.16	19.16	16.82	7.48	8.88
Greece–1	14.24	17.80	23.15	11.57	17.80	7.72	7.72
Greece–2	13.49	17.21	17.67	17.21	14.42	13.02	6.98
Italy	11.11	17.59	13.89	12.96	25.00	11.11	8.33
Japan	13.63	19.86	22.17	11.32	17.09	10.39	5.54
Lithuania	14.84	20.55	18.95	11.87	20.09	9.13	4.57
Mexico	15.36	18.43	24.23	6.83	22.87	8.87	3.41
New Zealand	10.64	13.68	18.84	15.50	22.80	10.33	8.21
Poland	12.00	15.50	21.50	15.00	18.00	12.00	6.00
Portugal	16.42	20.23	17.60	9.38	21.99	7.04	7.33
Republic of Serbia	14.96	22.44	19.70	12.22	16.71	7.23	6.73
Russia	12.77	19.39	22.38	12.21	17.81	10.09	5.36
Spain	12.19	19.66	22.80	11.40	19.27	9.83	4.85
Sweden	15.08	19.08	21.54	9.85	20.62	8.00	5.85
Turkey	13.05	16.63	23.37	11.58	17.68	9.68	8.00
UK	10.06	15.08	23.46	17.32	18.44	6.70	8.94
USA	14.06	15.48	22.24	11.21	17.97	12.10	6.94
Total sample	13.81	18.94	21.30	11.48	19.38	9.17	5.92

Stage 2: Specification of behavioral strategies for each TP profile. We used a combination of methods: 1) analysis of the plethora of available correlational studies on TP, 2) expert evaluations and 3) focus groups to get a qualitative description of a typical representative of each TP cluster, their most probable behavioral strategy in a social dilemma and the outcome of their interaction with other group members^ii^. As a result, we developed seven behavioral portraits:

Type 1: *Opportunistic*. This personality scores high on PN, PF and PH, moderately high on PP and moderately on FU. Type 1 initially would be *defector* (D) and won’t follow the established rules of energy consumption and will try to convert others to become D. Quite aggressive, confronts the rules, often argues, not very social. Possible character in the village: hard worker, but drunkard.

Type 2: *Orderly*. Such a person has moderate FU orientation, is moderately low on PH and PP, and low on PN and PF. Type 2 is initially *cooperator* (C) and will try to convince others into cooperation or following the established rules of energy consumption; S/he polices defectors 50% of the time, but doesn’t like to do it openly. Very organized, gets annoyed if somebody else is not, inflexible. Possible character in the village: a boring schoolteacher or a clerk at the shop.

Type 3: *Laborious*. Such a person is moderately high on PP and FU, moderate on PN and PF, and moderately low on PH. Type 3 would be a younger adult, goal oriented, focused and more innovative than the administration of the village, very social. Type 3 is initially C and tries to convince D to become a C. Possible character in the village: assistant nurse of the veterinarian who wants to be a doctor.

Type 4: S*teadfast*. Such a person is moderately high on PP and PH, moderate on PF, and moderately low on PN and FU. Type 4 is initially D; however, if they meet C, are policed or offered to be defector, s/he can be C for a certain period. Active in sports, energetic, enjoys life, spontaneous. Possible character in the village: someone retired, but still working for the community.

Type 5: *Precarious*. Such a person is moderately high on PH and PF, moderate on PN, and moderately low on PP and FU. Type 5 is initially D and keeps to it, unless C is a majority. Quite young (aged 25–30), unemployed, anxious, destructive health behaviors, risk-taker, sensation seeker.

Type 6: *Traumatized*. Such a person is moderately high on PN and PF, moderately low on PH and FU, very low on PP. Type 6 is initially can be either C or D with the same likelihood of either. Depressed, has to cope with many negative events in their past, problems with communication. Possible character in the village: war veteran.

Type 7: *Committed*. Such a person is high on FU, moderately high on PN, and moderately low on PH, PP and PF. Type 7 is initially C, will keep being C and will police others. Respectful, knowledgeable, perfectionist. Possible character in the village: Policing, administrative person.

Stage 3: Inspired by Lewin’s hypothesis that behavior (B) is a function of the person (P) and of his/her environment (E): B = F (P, E) [21], we created an agent-based model in a well-mixed population, assuming that each agent with a specific personality type is faced with a binary choice, either to cooperate or to defect.

Our main aim within this study was to keep the model as realistic as possible without making it overly complex. We did background research on the actual village, where the snowfall occurred (Huizinge, Netherlands). We found out that the population of the village was around 150 people; the village had 2 milk farms, a church, and a graveyard. We found out the type of generator used and the amount of electricity needed for roughly 100 households. Prior to building the actual simulation, we developed a face-to-face game, in which different roles were assigned to participants, such as: Mayor / Village Official, Mother/Father with two young kids, Teenage boy/girl, Octogenarian/War Veteran, Butcher, Priest, Blacksmith, Milk Farmer, Graveyard keeper/Descendant of the Alchemist, Postman, and Owner of the grocery store. The roles combined social roles that can be found in such a village with psychological characteristics of the TP profiles developed earlier.

Using the insights from the face-to-face game, we modeled a village populated by 150 agents. The original situation happened in the late 1970’s and in a small village, thus, we assumed that in such a setting all the villagers knew each other and their interaction was homogeneous. The well-mixed population design reflecting this assumption (homogeneous network structure where everyone is connected to everyone else with equal strength) was chosen for this model. Each agent was initially assigned one of the seven personality (TP) profiles associated with a set of rules describing their behavioral choice (to cooperate or defect). We used the obtained distribution of personality profiles (see Table 1) in order to “populate” our modeled village. The simulation proceeded by assuming that every agent at each time step (‘day’) made the decision of cooperating or defecting, based on his/her personality (TP) and environmental effects.

Initially, we defined the environmental effects as consisting of both physical components (the distance from the generator, the number of neighbors visible to each agent, etc.) and social components (interaction between the agents, their personal networks, degree of satisfaction with the existing rules of energy use, observation of neighbors’ behavior, social comparisons, etc.). For simplicity reasons we did not include the physical components in this model and decided to limit the environmental effects to the knowledge of the decision made at the previous step by the majority and to random interaction with other agents that can change individual behavior.

There were three types of interaction in our model: offering/being offered to cooperate, tempting/being tempted to defect, or policing/being policed for not following the rules. Policing is an external method of social control. Offering and tempting are internal (originating in one’s own motivation); these interactions are symmetrical, but directed in opposite ways (one invites to cooperate, and is tempted to defect). The model included two control parameters. When agents with non-cooperative TPs (opportunistic, steadfast, precarious, traumatized) were policed, they changed their behavior to cooperation with probability *h* and could sustain cooperation for *d* days, unless temped by defectors. In a real-life setting, the effect of being policed may depend on an individual’s TP profile. However, for simplicity we assumed that the effect of policing on being cooperative was only related to the control parameters, meaning the policing strength was the same for all TPs. Using control parameters *h* and *d*, we could emulate the situation in the above story and investigate how the policing strength affected the social dilemma. The agent interaction rules generated at Stage 2 are presented in Table 2.

**Table 2:** Initial conditions and interaction rules for agents in the model according to their personality types.

TP profile	Initial condition	C meets C	C meets D	D meets C	D meets D	If policed	If offered to be C	If tempted to be D
1	D	C	D	D (tempting to be D)	D	h% to be C for d days	NA	D
2	C	C	C (offering to be C, 50% of policing)	NA	NA	NA	NA	NA
3	C	C	C (50% of offering to be C)	NA	NA	NA	NA	NA
4	D	C	D	C for d days	D	h% to be C for d days	C for d days	D
5	D	C	C, but D if D is majority	D, but C if C is majority	D	h% to be C for d days if C is majority	C if C is majority	D if D is majority
6	50% C50% D	C	50% of becoming D	50% of becoming C	D	h% of becoming C	50% of becoming C	50% of becoming D
7	C	C	C_(policing)	NA	NA	NA	NA	NA

Note: C—cooperate, D—defect, h is the probability of cooperating (for personality types 1,4,5,6) for d days; percentages denote probabilities of respective changes.

To summarize, we address the issue of individual diversity in our model, a feature typically ignored in other simulation studies [12]. We understand and include the diversity in our model in the following ways: 1) by having agents with different behavior strategies (individual differences), 2) by including specific, non-uniform distributions of agents with different behavioral strategies, 3) by taking into account the diversity of possible interactions between the agents (offering/being offered to cooperate, tempting/being tempted to defect, or policing/being policed for not following the rules), and 4) by modeling the diverse outcomes of those interactions (either adopting a proposed strategy or not, imposing one’s own strategy, etc; the outcome of an interaction depending on the types of both agents meeting in a particular encounter).

## Results

**Fig 2:**
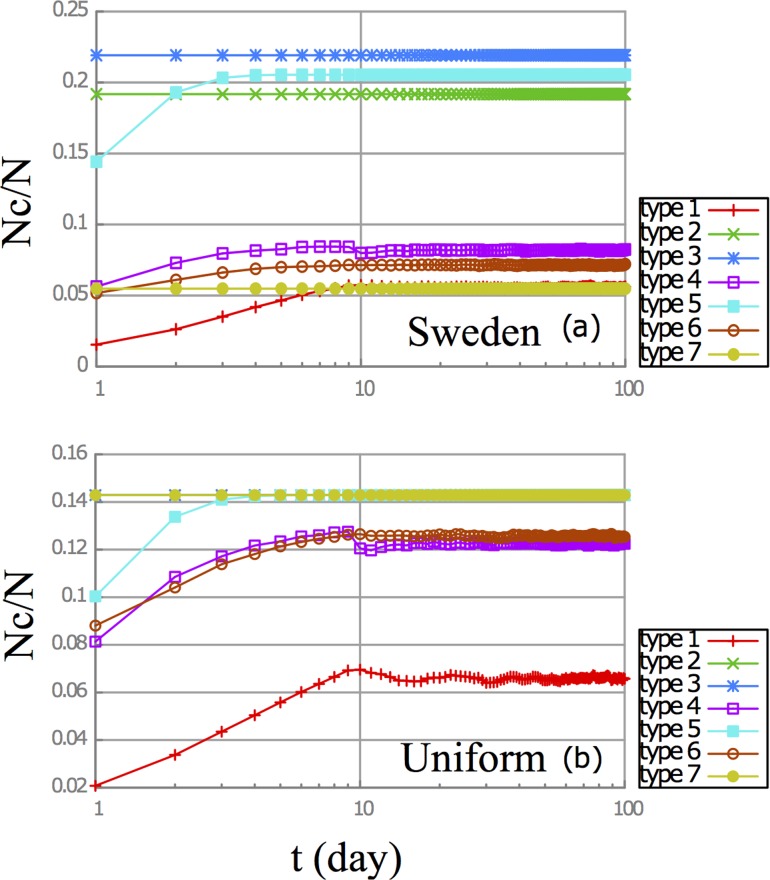
Daily behavior of seven personality types (top: Sweden, bottom: uniform distribution). Horizontal axis represents time (on a logarithmic scale), vertical axis represents percentage of cooperators. Note that for the uniform distribution the behavior of personalities 2, 3 and 7 are exactly similar.

To explore the behavioral patterns exhibited by each of the seven personality types, we ran the simulation for 1,000 discrete time steps (‘days’). The state of the system did not exhibit any qualitative changes as the number of days increased, and for reasons of simplicity the results of the first 100 days of simulation were used (see Fig. 2). The control parameters, *h* and *d*, were set to 0.50 and 10 days respectively. In the course of their interaction in time, different personalities produced different percentages of cooperators (Fig. 2, top). The village with a uniform distribution of seven personality types was used as a null model (Fig. 2, bottom)^iii^. In this simulation four personality types exhibited non-cooperative behavioral patterns, namely, types 1 (opportunistic), 4 (steadfast), 5 (precarious), and 6 (traumatized).

**Fig 3:**
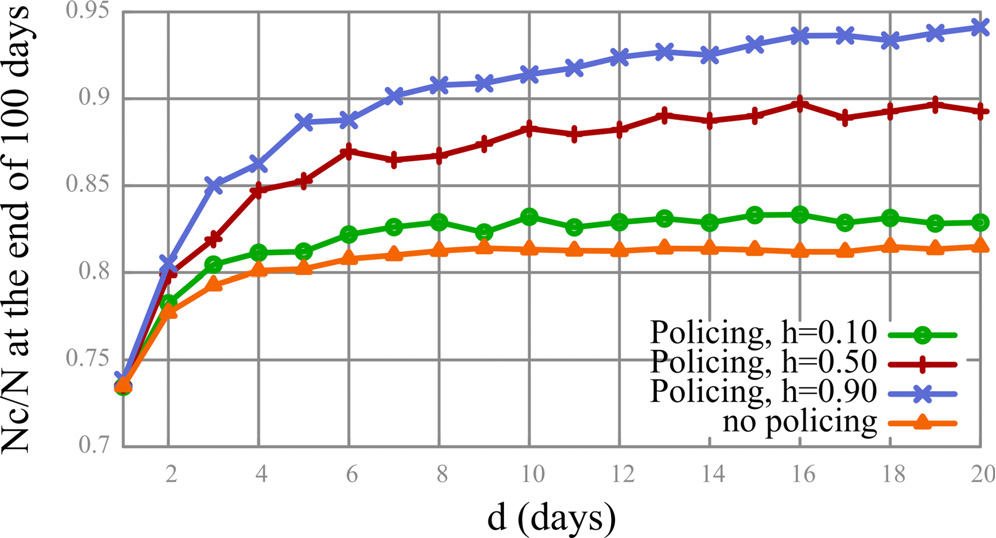
The effects of three different levels of policing on the percentage of cooperators. Horizontal axis represents different values of *d*.

Next, we looked into the impact of policing on behavior of non-cooperative types. We were interested in the degree to which environmental factors (policing) could affect the overall evolution of cooperation in a given situation. The results (see Fig. 3) showed that setting the probability of a non-cooperative type to become a cooperator when policed (parameter *h*) to 0.10 only marginally improved the situation in terms of the total number of cooperators. When *h* was set at 0.50, its impact on the cooperation outcome increased more strongly and noticeably, compared to an increase from 0 to 0.10 and to a further increase from 0.50 to 0.90. This suggested that when the agents were at least 50% inclined to become cooperators as a result of interaction, policing had a pronounced effect on the overall situation. We set the probability of becoming a cooperator when policed at 50% (*h* = 0.50) for the rest of the simulations.

**Fig 4:**
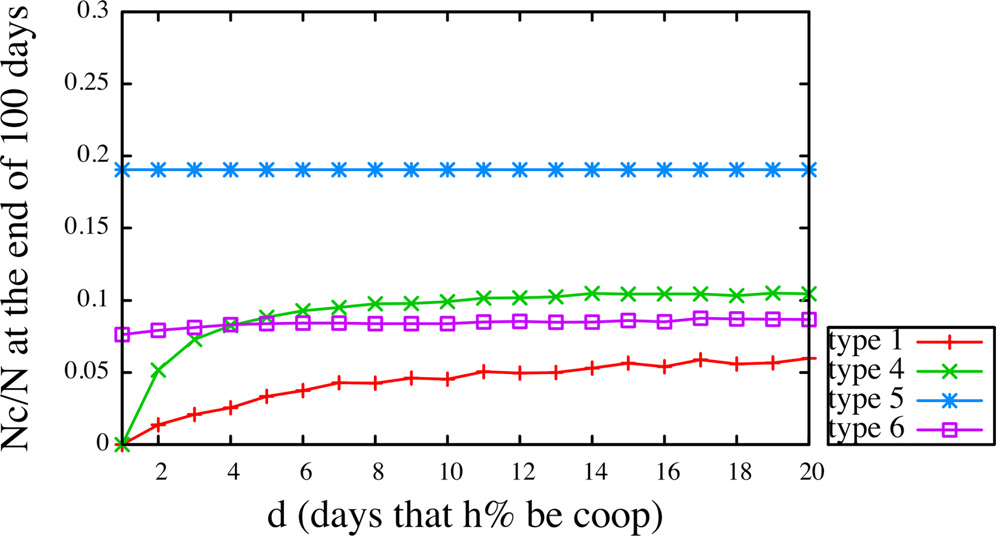
The learning curve for non-cooperative personalities in Sweden. We fix *h* = 0.50 and simulate the model for various *d* to see how the outcome of cooperation will improve for non-cooperative personalities.

Further, we explored the effect of habit, or the number of days for which the non-cooperative personality types (types 1, 4, 5 and 6) would adopt the cooperative strategy (in the case of a learning intervention, how likely and for how long they would sustain the effect). Fig. 4 shows that in Sweden (other countries presented in S2 Text, S1–S4 Figs.) type 6 (traumatized) remained stable and fluctuated little, depending on the chances of interacting with cooperators. Type 4 (steadfast) showed the strongest improvement, but needed to keep the cooperative strategy for at least three days to reach the level of type 6. The most non-cooperative type 1 (opportunistic) made the slowest progress, taking at least ten days before there was a noticeable effect on the overall outcome. These analyses reflect the individual differences between the types in their learning strategies and dynamics of adoption of cooperative behavior pattern.

**Fig 5:**
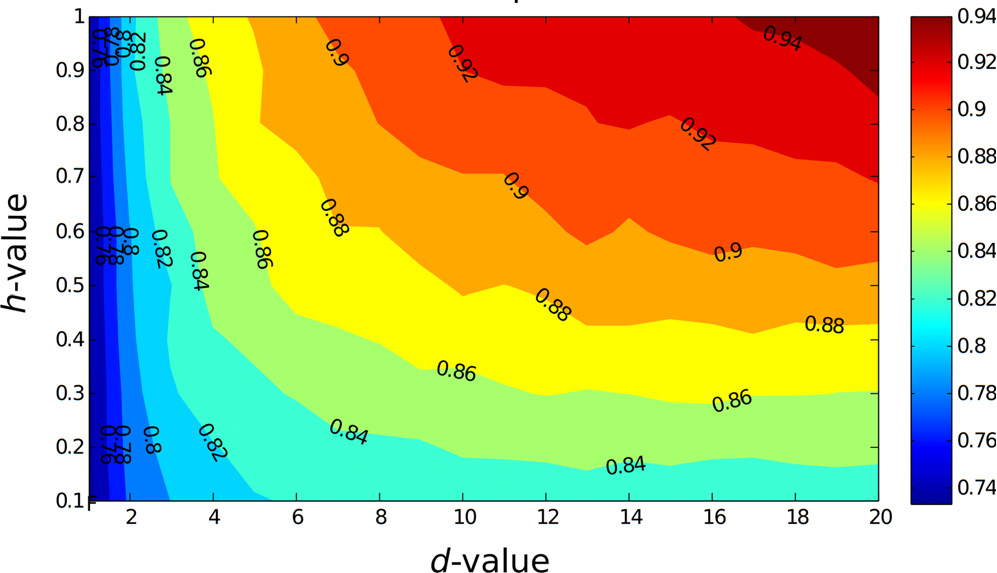
Fraction of overall cooperators in Sweden. The contour plot of overall percentage of cooperators after 100 days depending on the control parameters *h* (policing efficiency) and *d* (days behavior changes). Horizontal axis shows value of *d* and vertical axis represents *h*.

Then we examined the interplay between *h* (tendency to be cooperative) and *d* (duration of being cooperative). Fig. 5 shows the total fraction of cooperators in Sweden depending on the values of *d* (x-axis) and *h* (y-axis). By increasing the *d*- and *h*- values, the overall percentage of cooperators after 100 days increased markedly. This visual representation of the behavior of the complex system allowed us to investigate the non-linear interactions between the parameters. For instance, if the non-cooperative types were to adopt a cooperative behavior strategy for six days, and at the same time the tendency to cooperate increased from 30% to 70%, the overall percentage of cooperators increased from 82% to 86%. When the personal tendency to be a cooperator among the population was as low as 30% (by setting *h* to. 3), the educational effect reached its peak at six days and its further increase did not influence the proportion of cooperators, in contrast to higher levels of *h*. The personality type distributions in different countries exhibited different dynamical patterns of change in the fraction of overall cooperators (see S3 Text, S5–S9 Figs.).

Our model did not include any explicit penalty for non-cooperative behavior detected by the policing agents. However, the probabilities *h* and *d* model these processes in an indirect way. For instance, when the penalty is high and unavoidable, we can expect agents to feel more fear. As a result, they would stop non-cooperative behavior with a higher probability (*h*) and would keep cooperating for a longer time (*d*), resulting in an overall improvement of cooperation within the social system. Thus, overall cooperation is improved as parameters *h* and *d* increase (see Fig. 5). An interesting point in our results is the existence of an optimal ratio between *h* and *d*. For a given *h*, the percentage of cooperators is saturated after a certain *d* days. Because the parameters *h* and *d* are associated, both need to increase to improve the overall cooperation level.

**Fig 6:**
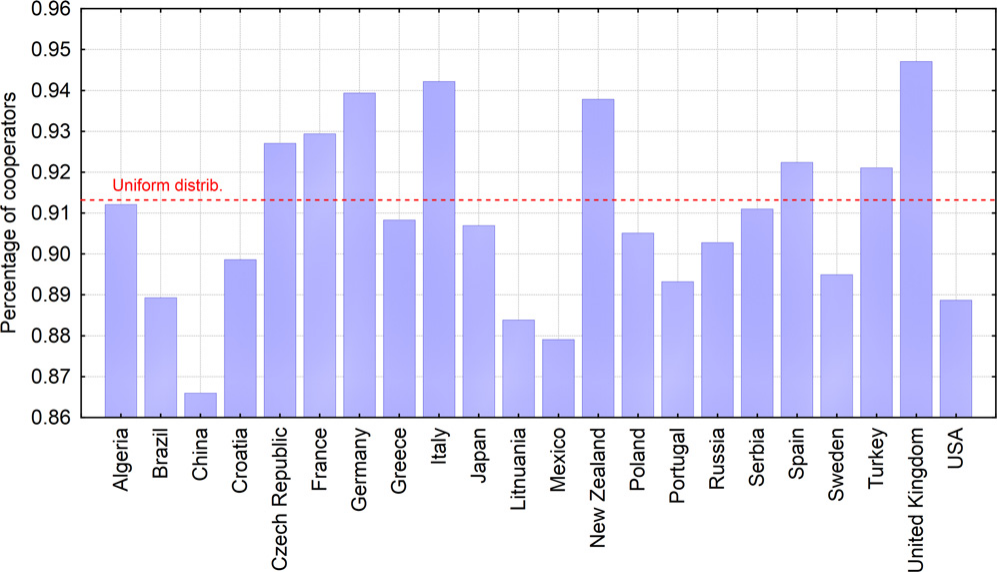
Cooperation index across countries. The control parameter in the model are fixed for *h* = 0.50 and *d* = 20. The values are reported according to the fitting results based on the equation 1.

To get the best estimation of the equilibrium level of cooperation, we fitted our simulation results into the following function:

$$y(d)=C+B\exp\left(-\frac{d}{F}\right)$$ (1)

where the value of *d* (days of cooperation for non-cooperatives) was set to.0 < *d* ≤ 20.The fitting parameters are *B*, *F* and *C*. The parameter *B* determines the initial value of the curve and parameter *F* determines the steep of the curve. Parameter *C* is the equilibrium level that determines the final stage of cooperation as *d* goes to infinity. Fig. 6 reports the equilibrium level and presents a ranking of the 22 country samples based on the overall percentage of cooperators, or cooperation index, which depends on the distribution of the seven personality types in each given country.

To further validate our cooperation index, we investigated its associations with various socioeconomic and environmental performance indicators. Previous studies showed that economic system, level of general trust, political system, and religion could influence the level of cooperation in societies [36, 37]. We used the Human Development Index [38] (Human Development Report, 2011), six dimensions of national culture [39], the Environmental Performance Index (EPI) [40], and the Democracy Ranking [41]. Table 3 shows that the percentage of cooperators is positively associated with country-level human development, environmental performance, and the quality of democracy, as well as higher individualism and lower power distance (of the Hofstede cultural values). We also found a significant non-linear association with Uncertainty Avoidance (UA). The quadratic regression function fit the data well (F_2, 18_ = 7.45; p<0.01; R^2^ = .45), indicating an inverted U-shaped association (in countries with the highest UA the percentage of cooperators was medium, while countries with high and low percentage of cooperators had lower UA scores—see *SI*: S10 Fig.).

**Table 3:** Correlations between the cooperation index and socio-economic indicators (N = 22).

	1	2	3	4	5	6	7	8	9	10
1. Cooperation index		.48*	.62*	.48*	-.43*	.55*	.23	.03	.04	.03
2. Human Development Index			.87*	.87*	-.73*	.74*	.14	-.15	.07	.30
3. Environmental Performance Index				.86*	-.63*	.55*	.01	.03	-.01	.15
4. Democracy Index					-.80*	.71*	-.09	-.18	-.23	.33
5. Power Distance						-.78*	.00	.54*	.08	-.36
6. Individualism / Collectivism							.08	-.50*	-.08	.31
7. Masculinity / Femininity								.06	.08	.10
8. Uncertainty Avoidance									-.11	-.34
9. Long-term orientation										-.54*
10. Indulgence / restraint										

Note: * p<.05. The number of countries was 21 for variables 4–10, as the data were not available for Algeria.

## Discussion

This study aimed to create an agent-based dynamic model of human interaction with rules based on patterns of individual differences observed in empirical data on time perspective across cultures. We developed our personality-driven model based on qualitative analysis of realistic personality types shown in empirical research, rather than deriving them post-factum [13]. We distinguished between seven latent profiles of time perspective, the personality factor that is the basis for behavior strategy of individual agents, whose dynamic interaction results in the overall outcome of the situation. Based on these seven personality types, we ran a simulation in a well-mixed population with random sequential updating, introducing two types of interactions: social control (policing) and education or persuasion (offering and tempting), and modeling contextual effects.

The simulation results suggest that specific personality types are prone to have a cooperative or non-cooperative behavior strategy (TP profiles 1, 4, 5, and 6 were shown as non-cooperative). In different social dilemmas (such as using a common water resource, fishing, banking etc.) different critical percentages of cooperating people are required to avoid depletion of a shared resource and the simulation data we obtained may be extrapolated, modified or reproduced to suit different kinds of social dilemmas.

Further, we examined the impact of interventions: policing, a form of external social control, and the role of internal motivation and social responsibility (offering to be a cooperator). We demonstrated that in order for policing to take effect on the population, the agents in every interaction should be at least 50% prone to be a cooperator. This suggests that social control does not have a pronounced effect on promoting the cooperative behavior unless the agents are sufficiently motivated to cooperate. This finding suggests an explanation for the contradiction that we observe regarding Sweden, for example. Sweden has the same cooperation index value as Brazil and is lower than Russia (see Fig. 6). However, it is at the ninth place on the EPI while Brazil is 77th [42]. Swedes might not be the best cooperators, but they may be sufficiently motivated to follow the rules and to comply with the strong pro-environmental policies, which makes them well prepared for the uncertain future.

We also looked into the dynamics of cooperation as a result of educational interventions (persuasion). We modeled different reactions to interaction between the agents, based on their personality types, suggesting that the types are not equally likely to change their behavior as a result of social interaction. Our results imply that in a realistic situation interventions aimed at behavior change should take into account the existing differences in personality types and to address them accordingly (i.e., future-oriented language might only be effective with future-oriented individuals, whereas those present-oriented would be more likely to respond to gamified interventions, and the interventions targeting the past-oriented would be more effective if they invoke tradition-based contexts).

We showed that the empirically observed differences in the distribution of TP personality types across countries had a prominent effect on the level of equilibrium in the resulting cooperative behavior. We believe that this result has important implications for the sampling procedures in future research. In some cases results may be skewed due to the under-representation of certain personality types existing in a given population.

Using the simulation outcomes, we developed an index of overall cooperation in a particular country, which we validated against cultural and economic indicators, such as the HDI, dimensions of national culture, EPI and Democracy Ranking. The resulting statistically significant associations provide evidence in favor of validity of our simulation procedure and of our assumptions about the behavior of agents with different TP types.

We can see interesting differences in the level of cooperation between the countries in our simulation. China, Mexico, Lithuania, Brazil, and Russia, countries with low levels of cooperation, have a young democracy or no democracy. On the other end of the continuum, we see higher cooperation in countries with a long history of democracy, such as France, New Zealand, Germany, and the UK. The possibilities for generalization are limited, as our country sample includes mostly Western and developed countries (mean HDI is 0.82). However, the results align well with the idea that cooperation (facilitated by specific TP profiles at the individual level) may form the basis for social development. Our results suggest that cooperation might be stronger in individualistic cultures with low power distance, In these societies people look after themselves and their direct family only and have equal rights, their superiors are accessible and power is decentralized. A non-linear association with uncertainty avoidance suggests that societies highly conducive to cooperation generally tend to be moderately permissive of unorthodox behavior. The notable exceptions, such as the USA, Sweden, and China, suggest a possible interaction of uncertainty avoidance with other cultural factors (i.e. individualism). A larger country sample is needed to test this hypothesis.

Coming back to our example of the Huizinge village, we can see that the mechanisms of social cooperation (policing and persuasion) included in our model are rather peculiar to individualistic cultures [43]. Other potential ways of coping with a generator breakdown are possible in collectivistic cultures: for instance, families could move in together and sacrifice their privacy for the sake of comfort for all (by using the same amount of energy to heat up a smaller number of homes). Other possible solutions of the village energy crisis could be based on Ostrom’s principles [44]. For example, clear rules of energy consumption developed and agreed upon collectively (collective-choice arrangement), could have influenced individual behavior. When people feel more agency with respect to the developed rules, they follow them more responsibly [37]. In our simulation, which reflects the norms and values of industrialized Western societies and the reality of surveillance society, we show that without the appointed policing villager (or monitoring principle in terms of Ostrom [44]), who points out transgressors on a regular basis, the cooperation fails. We were hoping, however, to find such conditions, under which meaningful actions of the villagers would start to occur, when the regulation would start from within, if it were at all possible. We were hoping to find an alternative solution to this particular critical situation.

Perhaps the main limitation of the study is the gap between self-report data on TP and the behavior of agents. It is quite possible that in a real-life situation other mechanisms we did not consider for inclusion in the model could influence the outcome of social interaction. We propose to see the present study as a case example showing the way agent-based modeling can be applied to investigate the effects of individual differences on the dynamics of evolution of complex social systems. This inter-disciplinary approach to social simulation can be adopted to explain the possible causes of global environmental issues and to predict their possible outcomes.

Time perspective is a dynamic process related to demographic and socioeconomic situations; therefore, the results may vary in different times and countries. Clearly, we need more empirical studies of the way time orientation manifests itself directly in people’s behavior and of the extent of influence people have over each other’s behavior. Investigating the effects of the structure and dynamics of the network of interactions on the cooperation with respect to individual differences [45] could be a promising future research avenue.

Another interesting extension of our work would be to elaborate the environmental dimension further and to include a realistic social network structure between the agents (such as the small-world or scale-free structures), as well as to include other parameters of the environment we considered (such as observing the behavior of neighbors, the role played by the agent in the network, participation in the creation of rules, degree of satisfaction with the rules, etc.). Additionally, it would be of interest to extend the model to a larger population with a mix of villages and cities, where the network topology would differ.

In summary, we demonstrate the link between personal TP profile, type of behavior strategy and interaction dynamics between different types in a critical situation. This approach can be used in resource management for scenario planning and developing risk management strategies. Our results can help better understand why similar policies or educational programs may work well in one place but don’t have a strong effect in another. We demonstrate the importance of considering the personality factor (individual differences) for sustainability policy makers, suggesting that policies that take into account the individual diversity may be more effective than the ‘one fits all’ approach. Our results also demonstrate that having strong and restrictive policies is not enough. Each member of the population has to be also motivated to follow those policies.

Our work shows that a combination of empirical personality research with ABM offers a possibility of a deeper understanding of human behavior. It is a tool for more refined simulations, in situations where experiments are not feasible. Such an approach makes it easy to vary different parameters and to test a variety of hypotheses. It can provide a better understanding of the interaction dynamics and the way behavior strategies develop over time.

Our method of including diversity in behavioral strategies of agents is a generic one and can serve as a placeholder. We propose a general methodology for including country-level survey data into an agent-based model, and thus creating a personality-driven agent-based model. We took time perspective profiles as a convenient example at hand, but it could have been Myers-Briggs typology with 16 types. It would be of interest to simulate other well-studied personality differences, such as the dimensions of the Big Five. However, we would like to stress the importance of the non-uniform distribution of types. As we have seen in our model, the initial composition of the population has an effect on the overall outcome, which should be accounted for in future studies on cooperation.

To the best of our knowledge, this study is the first attempt to link the heterogeneity of the individuals in terms of their personality (empirically shown individual differences in time perspective) with social interactions and the emergence of cooperation. We hope that our interdisciplinary exercise will open the way for future, more complex simulation models based on rigorous analysis of existing research data, including not only an interplay of agents with individual differences, but also the effects of social factors (rules, norms, values) and mechanisms that link individual-level and group-level phenomena.

### Endnotes

- This manuscript is based on data collected in 22 countries with the Zimbardo Time Perspective Inventory, under the supervision of Anna Sircova, with Fons Van de Vijver and Philip G. Zimbardo
- The experts (seven PhD students in Psychology in two consecutive meetings) were provided with a graphic representation of each cluster/type, a summary of the existing associations between TP factors and other variables (list presented in S1 Text) and the description of the village situation. They were asked to describe the most probable behavior of a specific type in the social dilemma based on the prevalence of different TPs and correlational results associated with it.
- The complete simulation was performed for all countries. The data for Sweden are presented in more detail, as this is the country where most of the authors were affiliated at the time of the study. Results for individual countries are presented in S2 Text, S1–S4 Figs., S3 Text, S5–S9 Figs. The cross-cultural comparisons are discussed where applicable.

## Supporting Information

S1 Fig The learning curve for non-cooperative personalities. We fix *h* = 0.50 and simulate the model for various *d* to see how the outcome of cooperation will improve for non-cooperative personalities. The results are shown for personality distribution of Algeria, Brazil, China, Croatia, Czech Republic and France.
(PDF)

S2 Fig The learning curve for non-cooperative personalities. We fix *h* = 0.50 and simulate the model for various *d* to see how the outcome of cooperation will improve for non-cooperative personalities. The results are shown for personality distribution of Germany, Greece, Italy, Japan, Lithuania and Mexico.
(PDF)

S3 Fig The learning curve for non-cooperative personalities. We fix *h* = 0.50 and simulate the model for various *d* to see how the outcome of cooperation will improve for non-cooperative personalities. The results are shown for personality distribution of New Zeland, Poland, Portugal, Serbia, Russia and Spain.
(PDF)

S4 Fig The learning curve for non-cooperative personalities. We fix *h* = 0.50 and simulate the model for various *d* to see how the outcome of cooperation will improve for non-cooperative personalities. The results are shown for personality distribution of Sweden, Turkey, UK and USA.
(PDF)

S5 Fig Fraction of overall cooperators index. The contour plot of overall percentage of cooperators after 100 days depending on the control parameters *h* (policing efficiency) and *d* (days behavior changes). Horizontal axis shows value of *d* and vertical axis represents *h*. The results are shown for personality distribution of Algeria, Brazil, China and Croatia.
(PDF)

S6 Fig Fraction of overall cooperators index. The contour plot of overall percentage of cooperators after 100 days depending on the control parameters *h* (policing efficiency) and *d* (days behavior changes). Horizontal axis shows value of *d* and vertical axis represents *h*. The results are shown for personality distribution of Czech Republic, France, Germany and Greece.
(PDF)

S7 Fig Fraction of overall cooperators index. The contour plot of overall percentage of cooperators after 100 days depending on the control parameters *h* (policing efficiency) and *d* (days behavior changes). Horizontal axis shows value of *d* and vertical axis represents *h*. The results are shown for personality distribution of Italy, Japan, Lithuania and Mexico.
(PDF)

S8 Fig Fraction of overall cooperators index. The contour plot of overall percentage of cooperators after 100 days depending on the control parameters *h* (policing efficiency) and *d* (days behavior changes). Horizontal axis shows value of *d* and vertical axis represents *h*. The results are shown for personality distribution of New Zeland, Poland, Portugal and Serbia.
(PDF)

S9 Fig Fraction of overall cooperators index. The contour plot of overall percentage of cooperators after 100 days depending on the control parameters *h* (policing efficiency) and *d* (days behavior changes). Horizontal axis shows value of *d* and vertical axis represents *h*. The results are shown for personality distribution of Russia, Spain, Sweden, Turkey, UK and USA.
(PDF)

S10 Fig Non-linear association of Hofstede’s Uncertainty Avoidance index with the cooperation index, resulting from simulation (100 days, *h* = 0.50, *d* = 20). (PDF)

S1 Text List of time perspective correlates used in the focus-group meetings for developing behavioral strategies. (PDF)

S2 Text Cooperation index for non-cooperative personalities as a function of day. (PDF)

S3 Text Overall cooperation index as a function of days of cooperation and probability of cooperation among non-cooperative personalities. (PDF)
