# Supplementary material for: Simulating Irrational Human Behavior to Prevent Resource Depletion
Source: PLoS One. 2015 Mar 11;10(3):e0117612. doi: 10.1371/journal.pone.0117612 (PMC4356575; doi:10.1371/journal.pone.0117612)

Figure S3. The learning curve for non-cooperative personalities. We fix $h = 0.50$ and simulate the model for various d to see how the outcome of cooperation will improve for non-cooperative personalities. The results are shown for personality distribution of New Zeland, Poland, Portugal, Serbia, Russia and Spain.

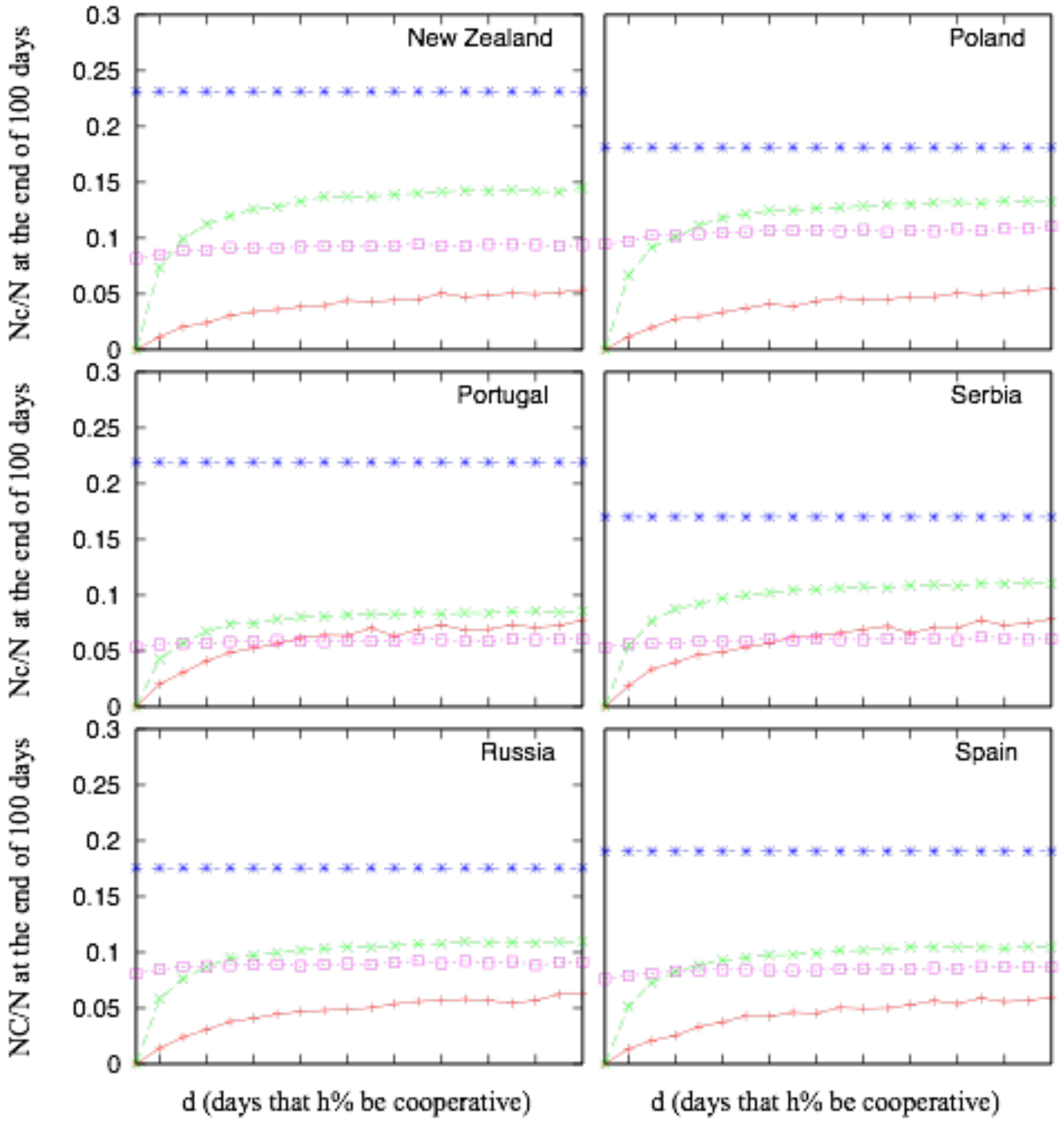

Supplement: S3 Fig — We fix h = 0.50 and simulate the model for various d to see how the outcome of cooperation will improve for non-cooperative personalities. The results are shown for personality distribution of New Zeland, Poland, Portugal, Serbia, Russia and Spain. (PDF) [file pone.0117612.s003.pdf]
