# Supplementary material for: Simulating Irrational Human Behavior to Prevent Resource Depletion
Source: PLoS One. 2015 Mar 11;10(3):e0117612. doi: 10.1371/journal.pone.0117612 (PMC4356575; doi:10.1371/journal.pone.0117612)

Figure S4. The learning curve for non-cooperative personalities. We fix h = 0.50 and simulate the model for various d to see how the outcome of cooperation will improve for non-cooperative personalities. The results are shown for personality distribution of Sweden, Turkey, UK and USA.

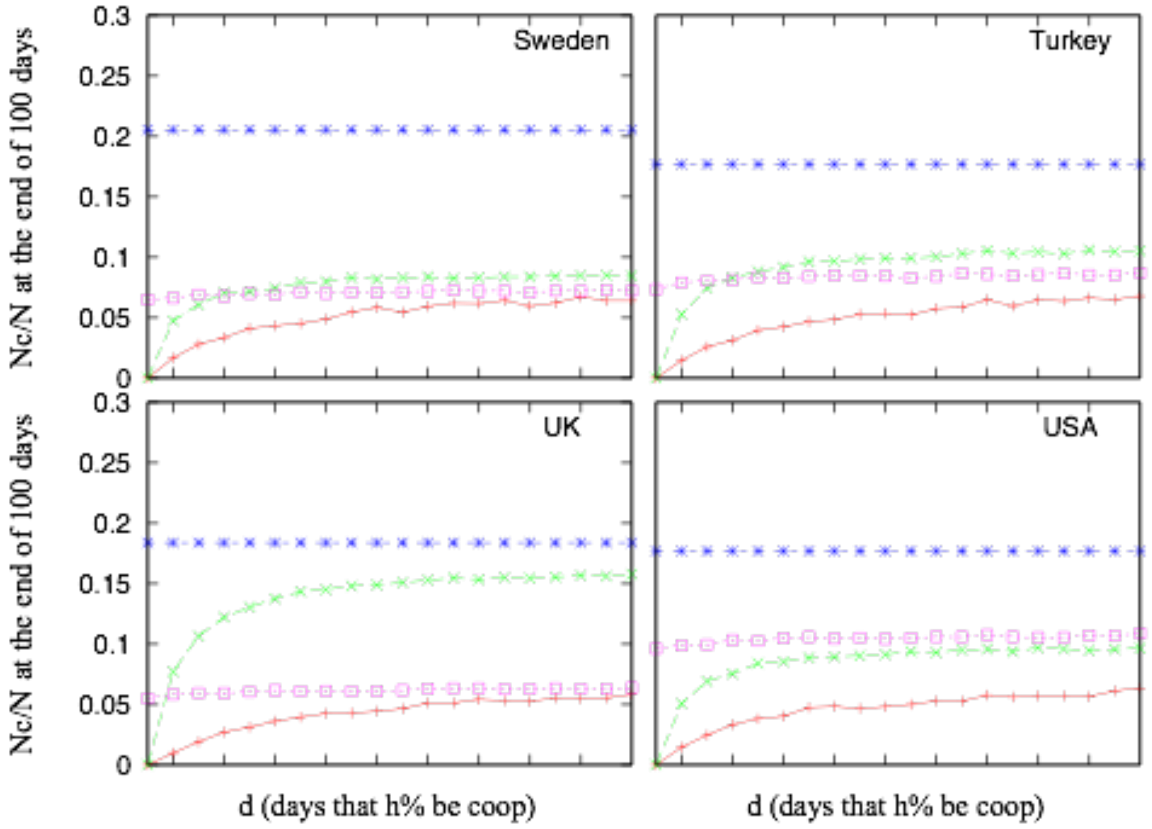

Supplement: S4 Fig — We fix h = 0.50 and simulate the model for various d to see how the outcome of cooperation will improve for non-cooperative personalities. The results are shown for personality distribution of Sweden, Turkey, UK and USA. (PDF) [file pone.0117612.s004.pdf]
