# Supplementary material for: Simulating Irrational Human Behavior to Prevent Resource Depletion
Source: PLoS One. 2015 Mar 11;10(3):e0117612. doi: 10.1371/journal.pone.0117612 (PMC4356575; doi:10.1371/journal.pone.0117612)

Figure S6. Fraction of overall cooperators index. The contour plot of overall percentage of cooperators after 100 days depending on the control parameters *h* (policing efficiency) and *d* (days behavior changes). Horizontal axis shows value of *d* and vertical axis represents *h*. The results are shown for personality distribution of Czech Republic, France, Germany and Greece.

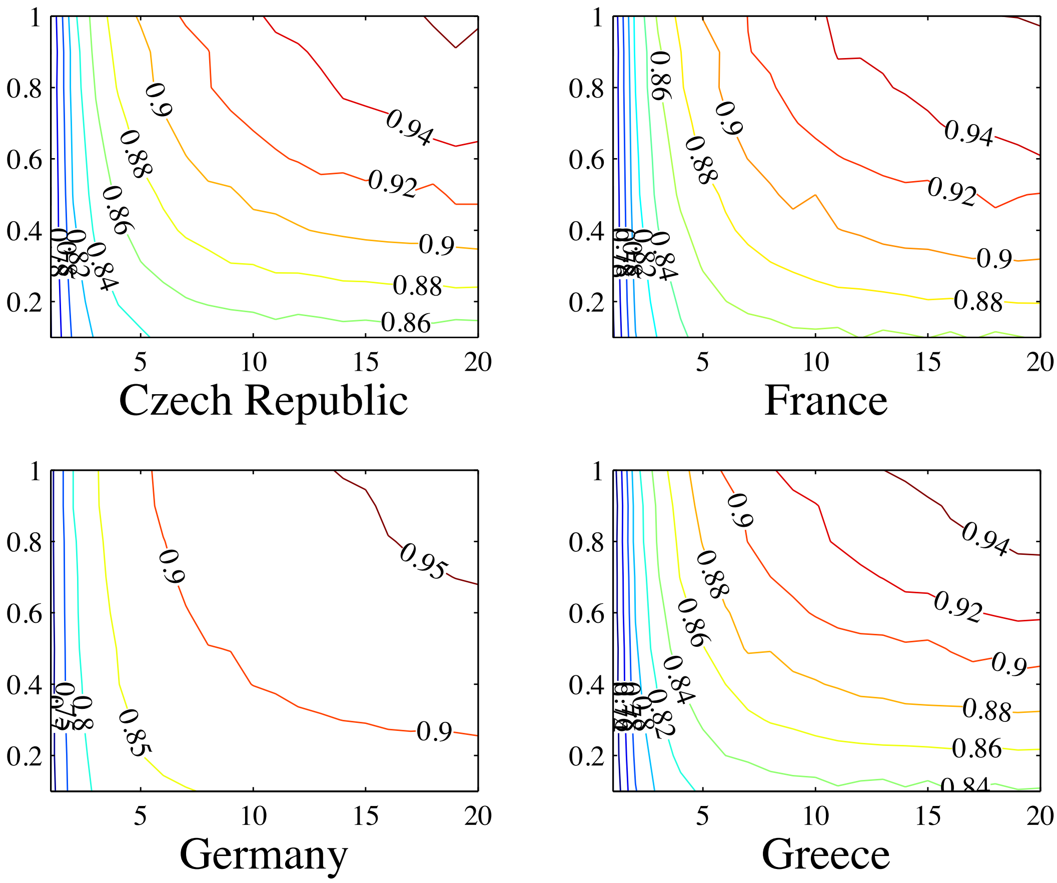

Supplement: S6 Fig — The contour plot of overall percentage of cooperators after 100 days depending on the control parameters h (policing efficiency) and d (days behavior changes). Horizontal axis shows value of d and vertical axis represents h. The results are shown for personality distribution of Czech Republic, France, Germany and Greece. (PDF) [file pone.0117612.s006.pdf]
