# Supplementary material for: Simulating Irrational Human Behavior to Prevent Resource Depletion
Source: PLoS One. 2015 Mar 11;10(3):e0117612. doi: 10.1371/journal.pone.0117612 (PMC4356575; doi:10.1371/journal.pone.0117612)

Figure S10. Non-linear association of Hofstede's Uncertainty Avoidance index with the cooperation index, resulting from simulation (100 days, *h*=0.50, *d*=20).

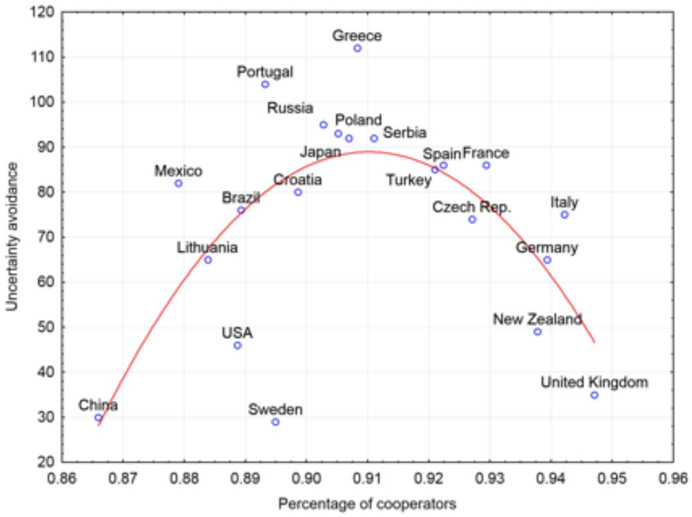

Supplement: S10 Fig — (PDF) [file pone.0117612.s010.pdf]
