# Supplementary material for: Simulating Irrational Human Behavior to Prevent Resource Depletion
Source: PLoS One. 2015 Mar 11;10(3):e0117612. doi: 10.1371/journal.pone.0117612 (PMC4356575; doi:10.1371/journal.pone.0117612)
Supplement: S1 Text — (PDF) [file pone.0117612.s011.pdf]

**Text S1. List of time perspective correlates used in the focus-group meetings for developing behavioral strategies**

Past Negative

- Depression
- Anxiety
- Unhappiness
- Low self-esteem
- Aggression
- Interpersonal relationships are minimal and unsatisfactory
- Not motivated to work for future rewards
- Have fewer close friends
- Exercise less, but like gambling
- Are less likely to have sex
- There are few aspects of their current life in which they report taking pleasure
- lack of emotional stability
- talk about their stress less frequently,
- experience more social conflict while coping,
- experience more negative emotions while coping
- Low risk-taking
- Pessimists
- Low on Conscientiousness, Extraversion and Agreeableness
- High on Neuroticism and Openness
- Locus of control: low on internality, high on chance and powerful others
- Increases with age in women

Past Positive

- Glowing, nostalgic, positive construction of the past
- Not aggressive
- Not depressed
- No anxiety
- High self-esteem
- Nostalgic focus on the past
- Introverted
- “better safe than sorry”
- Get involved in relationships with friends
- Shy
- Spiritual
- Have married parents
- Locus of control: high internality, low chance
- Optimism
- Cautious behavioral style
- Have fewer sex partners
- Consumed alcohol less often
- Take fewer risks
- Keep a clock prominently on their desks

- Happiness
- tend to cope with stressful situations more effectively
- enjoyed more social support, demonstrated more emotional growth coping and experience less social conflict while coping
- Positive correlation with *Right wing authoritarism*
- High on Conscientiousness, Extraversion and Agreeableness;
- Low on Neuroticism

Present Hedonistic

- Orientation toward present enjoyment, pleasure, and excitement, without sacrifices today for rewards tomorrow;
- Ego undercontrol
- Novelty seeking
- Sensation seeking
- No preference for consistency
- Used alcohol more
- Had unclear future goals
- Not religious
- Don't wear wristwatches
- Divorced parents, but communicate with families more often
- Highly energetic
- optimism
- less likely to practice safe sex
- Engage in many activities and a wide variety of sports
- more likely to take risk while driving
- more likely to have heated arguments than individuals lower in present hedonistic
- tend to talk about the stress of traumatic events but tended to use an avoidant coping style
- was related to decreased protective and increased risky health behaviors - *accent on pleasure, pleasurable health risking behavior*
- Locus of control: high on chance
- High Extraversion and Openness
- Low Conscientiousness and Neuroticism
- Decreases with age

Present Fatalistic

- No focused TP
- Belief that future is predestined and uninfluenced by individual actions
- Present must be borne with resignation because humans are at the whimsical mercy of "fate"
- Aggressive
- Anxious
- Depressed
- Don't consider the future consequences
- Intelligent people living in a generally optimistic environment that encourages a sense of personal efficacy, yet they do not believe that anything they do, or will do, is likely to make a difference in their lives.
- Tend to be dissatisfied with their present life and don't think it will improve.

- Lower GPA
- Didn't wish to that they had more time to spend with their friends
- Wanted to live shorter lives
- Many sexual partners, don't practice safe sex
- tend to talk less about their stress , cope with their stress less actively, avoid coping more frequentely
- *regular smokers, less seatbelt use. Fatalists – health-damaging behavior.*
- Locus of control: no internality, high on chance and powerful others
- Pessimists
- High Neuroticism
- Low Conscientiousness, Extraversion, Agreeableness and Openness
- Increases with age in women

Future

- Planning for and achievement of future goals
- Conscientiousness
- Consideration of future consequences
- Preference for consistency
- More hours spent studying per week
- No novelty seeking and sensation seeking
- Not depressed
- Highly organized and efficient
- Ambitious goal seekers, who felt pressed for time, but were willing to sacrifice present enjoyment to achieve their career objectives.
- Make "to do" lists, use a daily planner
- Wear a watch, balance their checkbook
- Have more order and structure in their lives
- Had clearly defined future goals
- Higher social class, Academic achievement, Higher motivation,
- Women with high F TP are more likely to have regular breast examination
- practice safe sex, eat healthy food more often,
- they avoid behaviors that are likely to be associated with negative consequences
- lower risk taking
- lower alcohol and substance abuse
- use slower speeds while driving
- Cope with traumatic events in a more active and analytic manner and showed less social conflict
- Negative correlation with *Social dominance orientation*
- pro-environmental behavior
- Increases with age
- Locus on control: high internality; low chance
