# Supplementary material for: Simulating Irrational Human Behavior to Prevent Resource Depletion
Source: PLoS One. 2015 Mar 11;10(3):e0117612. doi: 10.1371/journal.pone.0117612 (PMC4356575; doi:10.1371/journal.pone.0117612)
Supplement: S2 Text — (PDF) [file pone.0117612.s012.pdf]

Text S2. Cooperation index for non-cooperative personalities as a function of day

In the main text, we examined the cooperation index among non-cooperative personalities as a function of days that they cooperate (d) for Sweden as an example. Here we display the same results for all 22 countries that are mentioned and studied in the main text. The control parameter h is set to 0.5. The horizontal axis displays day and vertical axis displays fraction of cooperators.
