# Supplementary material for: Simulating Irrational Human Behavior to Prevent Resource Depletion
Source: PLoS One. 2015 Mar 11;10(3):e0117612. doi: 10.1371/journal.pone.0117612 (PMC4356575; doi:10.1371/journal.pone.0117612)
Supplement: S3 Text — (PDF) [file pone.0117612.s013.pdf]

Text S3. Overall cooperation index as a function of days of cooperation and probability of cooperation among non-cooperative personalities.

In the main text we examined the overall cooperation dependency on the two control parameters h(probability of cooperation among non-cooperative personalities) and d(days of cooperation among non-cooperative personalities) in the case of Sweden. Here we display the results for all 22 countries that are mentioned and studied in the main text. The horizontal axis displays parameter d(day) and vertical axis displays parameter h(cooperation probability), the contour lines display the overall fraction of cooperators.
